# A Selective Serotonin Reuptake Inhibitor, a Proton Pump Inhibitor, and Two Calcium Channel Blockers Inhibit *Candida albicans* Biofilms

**DOI:** 10.3390/microorganisms8050756

**Published:** 2020-05-18

**Authors:** Clarissa J. Nobile, Craig L. Ennis, Nairi Hartooni, Alexander D. Johnson, Matthew B. Lohse

**Affiliations:** 1 Department of Molecular and Cell Biology, School of Natural Sciences, University of California—Merced, Merced, CA 95343, USA; cnobile@ucmerced.edu (C.J.N.); cennis@ucmerced.edu (C.L.E.); 2 Quantitative and Systems Biology Graduate Program, University of California—Merced, Merced, CA 95343, USA; 3 Department of Microbiology and Immunology, University of California—San Francisco, San Francisco, CA 94158, USA; Nairi.Hartooni@ucsf.edu (N.H.); ajohnson@cgl.ucsf.edu (A.D.J.); 4 Department of Biochemistry and Biophysics, University of California—San Francisco, San Francisco, CA 94158, USA; 5 Department of Biology, BioSynesis, Inc., San Francisco, CA 94114, USA

**Keywords:** drug repurposing, high-throughput screens, biofilms, biofilm inhibition, biofilm disruption, *Candida albicans*, antimicrobial resistance, therapeutics, Pharmakon 1600 compound library

## Abstract

Biofilms formed by the human fungal pathogen *Candida albicans* are naturally resistant to many of the antifungal agents commonly used in the clinic. We screened a library containing 1600 clinically tested drug compounds to identify compounds that inhibit *C. albicans* biofilm formation. The compounds that emerged from the initial screen were validated in a secondary screen and then tested for (1) their abilities to disrupt mature biofilms and (2) for synergistic interactions with representatives of the three antifungal agents most commonly prescribed to treat *Candida* infections, fluconazole, amphotericin B, and caspofungin. Twenty compounds had antibiofilm activity in at least one of the secondary assays and several affected biofilms but, at the same concentration, had little or no effect on planktonic (suspension) growth of *C. albicans*. Two calcium channel blockers, a selective serotonin reuptake inhibitor, and an azole-based proton pump inhibitor were among the hits, suggesting that members of these three classes of drugs or their derivatives may be useful for treating *C. albicans* biofilm infections.

## 1. Introduction

*Candida albicans* is a member of the human microbiota that can asymptomatically colonize the skin, mouths, and gastrointestinal tracts of most individuals [1,2,3,4]. *C. albicans* is also one of the most common human fungal pathogens, where it can cause superficial dermal and mucosal infections in healthy individuals [1,5,6,7,8,9,10,11]. When a host’s immune system is compromised (e.g., in patients undergoing chemotherapy and in AIDS patients), *C. albicans* can give rise to disseminated bloodstream infections with mortality rates exceeding 40% [1,12,13,14,15].

An important virulence trait of *C. albicans* is its ability to form biofilms, structured communities of cells, on biotic and abiotic surfaces [1,4,9,16,17,18,19]. When mature, these biofilms contain a mixture of yeast, pseudohyphal, and hyphal cells surrounded by an extracellular matrix [1,3,17,18,19]. *C. albicans* biofilms form on mucosal surfaces, epithelial cell linings, and on implanted medical devices, including catheters, dentures, and heart valves [20,21]. These biofilms are typically resistant to antifungal drugs at concentrations normally effective against planktonic (suspension) cells, thus requiring higher drug concentrations in patients, which can cause side effects, such as liver or kidney damage [20,21,22,23,24,25]. *C. albicans* also forms complex polymicrobial biofilms with a wide range of bacteria, [26,27,28,29,30,31,32,33] whereby the biofilm structure provides a protected environment that can, for example, shield bacteria from environmental hazards (e.g., oxygen in the case of anaerobic bacteria) [34] or antibiotics [35,36,37]. The drug-resistant properties of *C. albicans* biofilms typically means that removal of biofilm-infected medical devices is the only treatment option for biofilm-based infections; however, device removal can be problematic when patients are already critically ill or when complicated surgical procedures are required (e.g., for a heart valve replacement) [20,38,39]. The development of new and alternative treatments effective against *C. albicans* biofilms is a priority considering the limitations of existing treatment options. Efforts in the field to address this medical need have included screens performed with novel compound libraries as well as screens of existing drugs that could be repurposed to target biofilms [40,41,42]. Several of these screens have been conducted in combination with existing antifungal agents (e.g., amphotericin B and miconazole) in order to identify synergistic effects [41,42].

Several experimental variables come into play when evaluating the ability of a compound to affect biofilms. For example, there are many techniques for quantifying biofilm formation. One common technique measures metabolic activity, as indicated by a colorimetric change resulting from reduction of the tetrazolium salt reagent XTT (or the closely related compound MTT) [43,44,45,46]. Another conceptionally similar approach uses the colorimetric change resulting from the reduction of Alamar Blue (also called resazurin or Cell Titer Blue) [47,48]. Both approaches rely on metabolic activity as a proxy for the extent of biofilm formation or for the number of viable cells remaining in the biofilm. If the reagent is unable to fully penetrate the biofilm structure or if there are large numbers of metabolically inactive but otherwise viable cells (e.g., persister cells), these types of assays can be difficult to interpret in certain situations (see, for example, Kuhn et al., 2003 and Honraet et al., 2008 for limitations of the XTT assay) [49,50]. The question of when, during the biofilm life cycle, a compound is evaluated can also affect results: is the compound tested for the ability to prevent the formation of a biofilm (inhibition) or for the ability to act against a mature biofilm (disruption)?

Here, we report a screen of the Pharmakon 1600 (MicroSource Discovery Systems, Inc.) library containing 1600 clinically tested drug compounds for those with *C. albicans* antibiofilm activity. This screen differs from the two previously reported screens of this library in three important aspects [41,42]. First, our primary screen and validating secondary screen focused on the ability of the compounds to prevent biofilm formation (inhibition), and an additional secondary screen focused on testing those initial “hits” for their abilities to disrupt mature biofilms. Second, the compounds were first screened for activity by themselves rather than in combination with or as a potentiating agent for an existing antifungal agent. Third, the effects on the biofilm were quantified using optical density biofilm assays, which directly measure biofilm formation [51,52], as opposed to measurements of metabolic activity [41,42]. Based on these screens, 43 compounds were further evaluated for synergy with the common antifungal drugs used in the clinic, fluconazole, amphotericin B, and caspofungin. Taken together, these screens revealed a number of compounds capable of inhibiting biofilm formation or disrupting mature biofilms by themselves or in combination with one or more existing antifungal agents.

## 2. Materials and Methods

### 2.1. Strains and Media

All assays used the previously reported SC5314-derived prototrophic a/α SNY425 strain [53]. Cells were cultured as previously described; in brief, cells were allowed to recover from glycerol stocks for two days at 30 °C on yeast extract peptone dextrose (YEPD) plates (2% Bacto peptone, 2% dextrose, 1% yeast extract, 2% agar). Overnight cultures for assays were grown for approximately 16 h at 30 °C in YEPD media (2% Bacto peptone, 2% dextrose, 1% yeast extract). Biofilm assays were performed in RPMI-1640 media (containing L-glutamine and lacking sodium bicarbonate, MP Biomedicals #0910601) supplemented with 34.5 g/L MOPS (Sigma, M3183) and adjusted to pH 7.0 with sodium hydroxide before sterilizing with a 0.22 µm filter [51,52].

### 2.2. Reagents

The Pharmakon 1600 compound library (MicroSource Discovery Systems, Inc. http://www.msdiscovery.com/index.html), which consists of 1600 clinically tested drug compounds approved for use in the United States and internationally, was obtained by UCSF’s Small Molecule Discovery Center (SMDC). Independent stocks of the 43 candidate compounds for further testing were obtained directly from MicroSource Discovery Systems, Inc. Working stocks of compounds were made at a concentration of 10 mM in DMSO (Sigma D2650).

### 2.3. Biofilm Assays

The high throughput adherence inhibition, sustained inhibition, and disruption variants of the standard optical density biofilm assay followed previously reported 384-well format protocols [51,52,54,55]. Compounds of interest, as well as the known antifungal agents for the combination assays, were added during the 90 min adherence step for the adherence optical density biofilm assay, at both the 90 min adherence and 24 h growth steps of the sustained inhibition optical density biofilm assay, and at the second 24 h growth step of the disruption optical density biofilm assay [51,52]. In brief, 1 µL of overnight culture was added to 90 µL media (or media with drug) in the well, giving a final OD_600_ = 0.15, or roughly 2 × 10^6^ cells/mL. Plates were sealed with Breathe-Easy sealing membranes (Diversified Biotech, BEM-1) and shaken at 37 °C for 90 min at 350 rpm in an ELMI (DTS-4) incubator. Media were then removed, wells were washed once with PBS, and fresh media (or media with drug) was then added back to wells. Plates were then resealed and shaken at 37 °C for 24 h at 350 rpm in an ELMI incubator. For inhibition assays, media were removed at this point and the absorbance (OD_600_) was determined on a Tecan Infinite M1000 Pro or a Tecan M200. For disruption assays, media were removed in groups of 6 to 12 wells and fresh media containing the compound of interest were carefully added back to the wells on the side of the well opposite the side from which media was removed. Plates were then resealed and shaken at 37 °C for a further 24 h at 350 rpm. Media were removed after this second 24-h growth step and the absorbance (OD_600_) was determined on a Tecan Infinite M1000 Pro or a Tecan M200.

The low throughput Adherence Inhibition variant of the Standard Optical Density Biofilm Assay followed the previously reported 96-well format protocol [51,52,54,55] with the following differences from the 384-well format protocol described above. Briefly, cells from the overnight culture were added to 200 µL media (or media with drug) to a final OD_600_ = 0.5, or roughly 1 × 10^7^ cells/mL. Plates were shaken at 250 rpm during the 90 min adherence and 24-h growth steps. The absorbance (OD_600_) was determined on a BioTek Epoch 2 taking the average of 21 reads per well.

### 2.4. High Throughput Adherence Inhibition Screen

**Figure 1:**
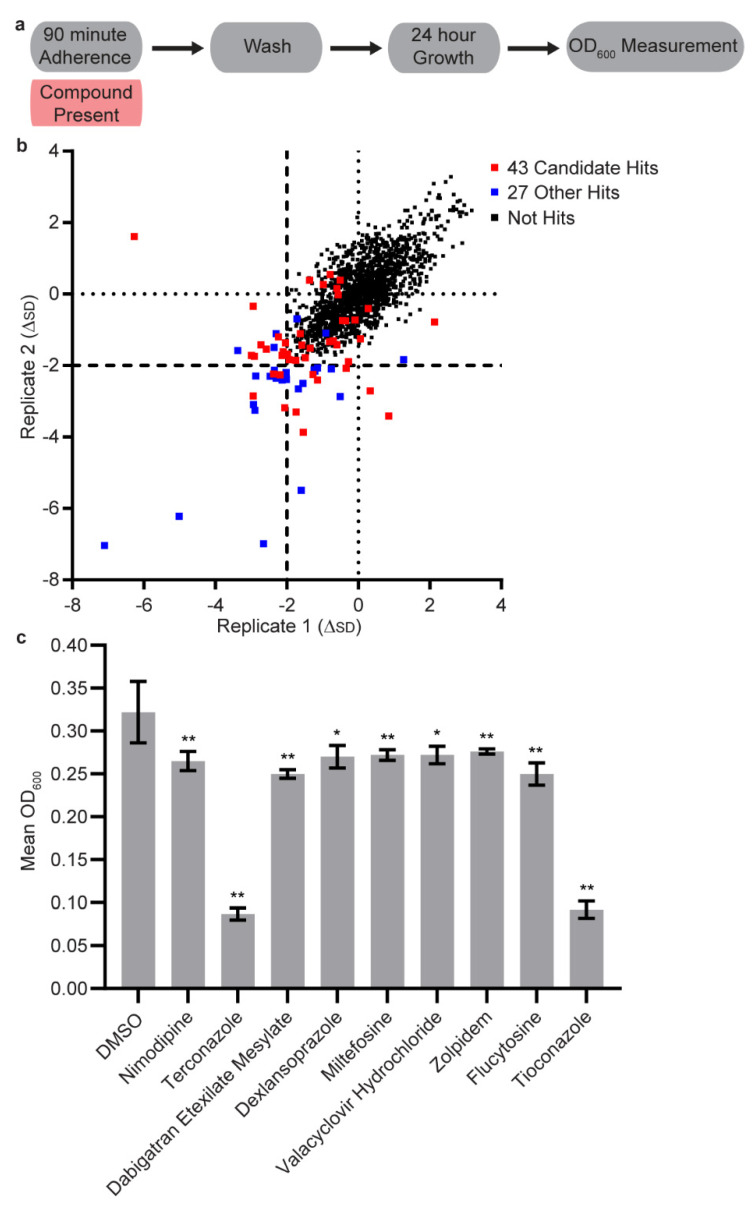
High-throughput screening of the Pharmakon 1600 compound library for the ability to inhibit *C. albicans* biofilm formation. (**a**) Overview of the adherence inhibition optical density biofilm assay used in these experiments. Compounds were included at a concentration of 10 µM during the 90-min adherence step but not in the 24-h growth step of in vitro biofilm formation. (**b**) Comparisons of the differences from the mean, in units of standard deviation, for each of the 1600 compounds in the two replicate assays. The 43 candidate hits that were pursued further are indicated in red. The 27 other hits that were not pursued, which consisted of a mixture of well-known antifungal agents, compounds intended for topical use, and compounds with high toxicity values (<100 mg/kg), are indicated in blue. All other compounds are indicated in black. On each axis, the dotted lines indicate no difference from the mean and the dashed lines indicate a threshold of two standard deviations below the mean. (**c**) Subset of the statistically significant hits at 40 µM from the adherence inhibition optical density biofilm assay. Mean OD_600_ readings with standard deviations are shown, significant differences from the DMSO solvent control as determined by Welch’s *t*-test (two-tailed, assuming unequal variance) with the Bonferroni correction are indicated for α = 0.05 (*) and α = 0.01 (**).

Two independent, high throughput screens were robotically performed using the complete Pharmakon 1600 compound library in the adherence inhibition biofilm assay on separate days. Compounds were added at 10 µM to a single well in both runs. Forty-five compounds had effects on biofilm formation that were at least two standard deviations below the mean in at least one of the experiments (17 compounds had this effect in both screens, 13 compounds only had this effect in the first screen, and 15 compounds only had this effect in the second screen) (Figure 1b). An additional 25 “promising” compounds that missed the two standard deviation threshold cutoff were selected for further study based on statistical analysis (B score less than −4) and a manual inspection of the data (Figure S1). The 70 compounds on these lists were then curated in order to eliminate compounds with high toxicities (less than 100 mg/kg), compounds intended for topical use, and most of the well-known antifungal agents (with the exception of a few included as positive controls). After culling the list according to these criteria, 43 candidates remained for further study. Four of these 43 compounds had effects on biofilm formation that were at least two standard deviations below the mean in both high throughput screens, 17 compounds had these effects in only one of the two screens, and the remaining 22 compounds were part of the “promising” compounds lists. Data for these screens can be found in File S1. A list of the 70 original compounds and a summary of the justifications for excluding those compounds not in the final set of 43 can be found in File S2.

### 2.5. Low Throughput Adherence Inhibition Screen

The 43 candidate compounds were tested individually at both 10 µM and 40 µM in the adherence inhibition optical density biofilm assay [51,52] in order to validate the results of the initial high throughput screen. For these stand-alone assays, the candidate compounds (and controls) were tested in groups of four wells, one set of four wells was used for each compound at each concentration, and the 43 compounds were split between two plates for each concentration (for a total of four plates). Each plate had one set of control wells (four wells, 16 total control wells across the four plates) containing equivalent volumes of DMSO to the experimental wells. For each experimental set of four wells, significance was evaluated versus all of the control wells from the four plates (16 total) by performing Welch’s *t*-test (two-tailed, assuming unequal variance). Further details on the statistical analyses for these experiments can be found in File S3. A summary of hits from these assays are included in File S4. Data and statistics for the 10 µM and 40 µM low throughput adherence inhibition optical density biofilm assays are compiled in File S5.

### 2.6. Sustained Inhibition and Disruption Testing

The 43 candidate compounds were tested individually at 40 µM in the sustained inhibition optical density biofilm assay and in the disruption optical density biofilm assay [51,52]. For these stand-alone assays, individual repeats of candidate compounds (and controls) were performed in groups of eight wells. Between two and four repeats (16 to 32 total wells) were used for each candidate compound. Each plate had four or five sets of control wells (32 or 40 total wells), containing equivalent volumes of DMSO to the experimental wells, located throughout the plate to reduce positional effects. For each experimental set of eight wells, significance was evaluated versus all of the control wells from the same plate by performing Welch’s *t*-test (two-tailed, assuming unequal variance). Further details on the statistical analyses for these experiments can be found in File S3. A summary of hits from these assays are included in File S4. Data and statistics for the stand-alone sustained inhibition and disruption optical density biofilm assays are compiled in File S5.

### 2.7. MBIC Determination

We determined the minimum biofilm inhibitory concentration (MBIC) of compounds that inhibited biofilm formation in our low throughput screens using the 384-well format of the sustained inhibition optical density biofilm assay [51,52]. Candidate compounds were diluted fourfold from a maximum concentration of 200 µM to a minimum concentration of 0.78 µM (five concentrations tested) and equivalent volumes of DMSO were used as loading controls for the compounds. Groups of eight wells were used for each candidate compound or control condition. Details on the statistical analyses for these experiments can be found in File S3. Data and statistics for the BIC sustained inhibition optical density biofilm assay are compiled in File S5.

### 2.8. MIC Determination

We determined the planktonic minimum inhibitory concentration (MIC) of compounds that inhibited biofilm formation or disrupted mature biofilms in our low throughput screens using a 96-well planktonic MIC assay [56,57,58]. In brief, these assays were performed at 30 °C in YEPD media. Compounds were diluted twofold from a maximum concentration of 200 µM to a minimum concentration of 0.2 µM (eleven concentrations tested). A DMSO dilution series was used as the loading control for the compounds and untreated wells were included on each plate. Two replicates on independent plates were performed for each compound. If there was not a clear reduction in growth at any concentration after 48 h of growth at 30 °C, the MIC is indicated as greater than 200 µM (the highest concentration tested). If a clear reduction in growth was observed for all concentrations tested after 48 h of growth at 30 °C, the MIC was indicated as less than or equal to 0.2 µM (the lowest concentration tested). Data for the planktonic MIC assays are compiled in File S6.

### 2.9. Combination Screening

The candidate compound plus known antifungal agent combination sustained inhibition and disruption optical density biofilm assays used the protocols described above with the following modifications. Candidate compounds were included at a concentration of 12.5 µM in both assays with the following exceptions: in the combination sustained inhibition assay terconazole, dabigatran etexilate mesylate, miltefosine, and tioconazole were included at 0.2 µM; dexlansoprazole was included at 0.8 µM; and chloroxine was included at 3 µM. In the combination disruption optical density biofilm assay, mefenamic acid was included at 3 µM. The sustained inhibition optical density biofilm assays used 1 µg/mL amphotericin B, 0.125 µg/mL caspofungin, or 256 µg/mL fluconazole. The disruption optical density biofilm assays used 2 µg/mL amphotericin B, 0.5 µg/mL caspofungin, or 256 µg/mL fluconazole. The sensitivity of SNY425 to amphotericin B, caspofungin, and fluconazole in our biofilm assays is reported in File S5.

For the combination assays, compounds (and controls) were again tested in groups of eight wells and two distinct groups of controls were included on each plate. The first set of controls contained wells with the candidate compound, but no known antifungal agent. The second set of controls contained wells with the known antifungal agent, but no candidate compound. In both cases, the concentration of candidate compound or known antifungal agent was the same as was used in the experimental wells. Controls were included for all candidate compounds and antifungal agents tested on a given plate. In general, a single set of eight wells was included for each experimental or control condition on a given plate. Details on the statistical analyses for these experiments can be found in File S3. A summary of hits from these assays are included in File S4. Data, statistics, and concentrations used for the combination sustained inhibition and disruption optical density biofilm assays are compiled in File S5.

## 3. Results

### 3.1. Initial Screen

We performed two independent high throughput screens of the Pharmakon 1600 compound library (MicroSource Discovery Systems, Inc.), which contains 1600 clinically tested drug compounds approved for use in the United States and internationally, for compounds with the ability to inhibit biofilm formation in the adherence inhibition optical density biofilm assay [51,52]. In this assay, compounds are added during the 90-min initial step of biofilm formation, then washed out (along with unadhered cells). The biofilm was then allowed to develop for 24 h in the absence of the compound (Figure 1a). Seventeen compounds had effects on biofilm formation that were at least two standard deviations below the mean in both replicate screens and an additional 28 compounds exhibited a similar phenotype in one but not both of the screens (File S1). To these 45 compounds, we added an additional 25 “promising” compounds manually selected based on noticeable effects that fell short of the two standard deviation threshold (a combination of compounds with a B score less than −4 and ones that were selected based on manual inspection of the data). From this list of 70 compounds, we eliminated most of those with high toxicities and we also removed some compounds with well-established antifungal activities. As controls, we included several known antifungals to give a total of 43 compounds that entered our secondary screens (Figure 1b and Figure S1, File S2). Four of these 43 compounds had effects on biofilm formation that were two standard deviations below the mean in both high throughput screens, while 17 compounds had had effects on biofilm formation that were two standard deviations below the mean in only one of the two screens. The remaining 22 compounds were part of the “promising” compounds lists. As will be described below, we carried out several different types of secondary screens with the goal of identifying compounds that might be especially effective in a particular setting. Because we do not know which in vitro assay best mimics the situation in vivo, we adopted this approach to maximize our chances of identifying useful compounds.

### 3.2. Secondary Screens

We evaluated the effects of the 43 candidate compounds in a low throughput version of the adherence inhibition optical density biofilm assay [51,52] at both 10 µM and 40 µM. In general, the strongest hits from the initial screen correlated well with this secondary screen, but most of the weaker hits (especially those with effects in only one of the two duplicate assays) did not pass the secondary test. Two compounds had effects at 10 µM (the known antifungals tioconazole and terconazole) and ten had effects at 40 µM (Figure 1c and Figure S2, File S4). The latter included both compounds that had an effect at 10 µM as well as the known antifungals flucytosine, chloroxine, and miltefosine. The remaining five compounds, which—to the best of our knowledge—have not been implicated in activity against *C. albicans* biofilms, include the calcium channel blocker nimodipine, the thrombin inhibitor dabigatran etexilate mesylate, the proton pump inhibitor dexlansoprazole, the viral DNA replication inhibitor valacyclovir hydrochloride, and the GABA receptor agonist zolpidem.

**Figure 2:**
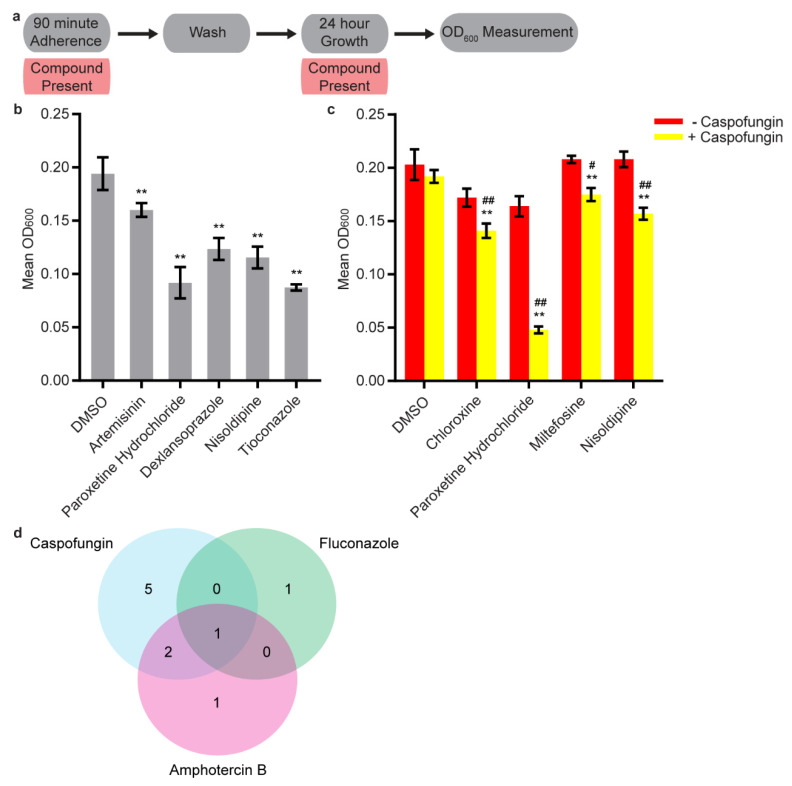
Thirteen candidate compounds inhibited biofilm formation by themselves or in combination with one or more known antifungal agents. (**a**) Overview of the experimental setup for the sustained inhibition optical density biofilm assay used for these experiments. Compounds were included during both the 90-min adherence step and the 24-h growth step of in vitro biofilm formation. (**b**) Subset of the statistically significant hits from the stand-alone sustained inhibition optical density biofilm assay. Mean OD_600_ readings with standard deviations are shown, significant differences from the DMSO solvent control as determined by Welch’s *t*-test (two-tailed, assuming unequal variance) with the Bonferroni correction are indicated for α = 0.05 (*) and α = 0.01 (**). Although a single repeat is shown, the indicated threshold was met by all of the repeats of each compound shown. (**c**) Subset of the statistically significant hits from the combination sustained inhibition optical density biofilm assays with caspofungin. For each compound, the wells with caspofungin (+ caspofungin) are indicated in yellow and wells without caspofungin (– caspofungin) are indicated in red. Mean OD_600_ readings with standard deviations are shown; significant differences from the compound without caspofungin control (e.g., chloroxine, − caspofungin), as determined by Welch’s *t*-test (two-tailed, assuming unequal variance) with the Bonferroni correction, are indicated for α = 0.05 (*) and α = 0.01 (**). Significant differences from the caspofungin without compound control (e.g., DMSO, + caspofungin), as determined by Welch’s *t*-test (two-tailed, assuming unequal variance) with the Bonferroni correction, are indicated for α = 0.05 (#) and α = 0.01 (##). In panels b and c, data within a chart are taken from the same plate on the same day. (**d**) Venn diagram illustrating the degree of overlap between the combination sustained inhibition screens with amphotericin B, caspofungin, and fluconazole.

We also evaluated the effects of our initial 43 candidate compounds in a different type of assay, the sustained inhibition optical density biofilm assay (Figure 2a). Here, compounds were added to media at 40 µM both during the 90-min adherence step (as was done in our initial screen) and during the subsequent 24-h growth step. Ten compounds had a consistent effect in this assay; among these were the known antifungal agents (serving as positive controls) tioconazole, terconazole, flucytosine, chloroxine, and miltefosine (Figure 2b and Figure S3a, File S4). Artemisinin also had an effect, a result consistent with a previous report that the related compound artesunate had an antibiofilm synergy with miconazole [42]. Among the new compounds identified as having activities against *C. albicans* biofilms are the calcium channel blocker nisoldipine, the selective serotonin reuptake inhibitor paroxetine hydrochloride, and the proton pump inhibitor dexlansoprazole (Figure 2b and Figure S3a, File S4). Six of the compounds with effects in this assay also had effects in the low throughput adherence inhibition assay at 40 µM (dexlansoprazole and the five known antifungal agents). We do not fully understand why some compounds showed significant inhibition in the adherence inhibition optical density biofilm assay but not in the sustained inhibition optical density biofilm assay, but the different assays may be sensitive to different compound parameters such as solubility, stability, and pH dependence.

To distinguish between biofilm-specific and general fungicidal or fungistatic effects, we determined the minimum biofilm inhibitory concentration (MBIC) of these ten compounds in the sustained inhibition assay and the minimum inhibitory concentration (MIC) for planktonic cells (Table 1). One of the new hits, dexlansoprazole, inhibited biofilm formation at low micromolar concentrations (3.1 µM, Table 1). Unlike the known antifungal agents, which also affected planktonic *C. albicans* cells at low micromolar concentrations, dexlansoprazole had a planktonic MIC of at least 200 µM. In other words, dexlansoprazole displayed biofilm-specific effects that did not extend to planktonic cells. We note that nisoldipine and paroxetine hydrochloride also had MBICs that were well below their planktonic MICs (MBICs of 50 µM; MICs of at least 200 µM, Table 1).

### 3.3. Synergy Screening

Given the previous reports suggesting antibiofilm synergies between known antifungal agents and certain drug classes, we next evaluated whether any of our 43 initial candidate compounds at low concentrations (12.5 µM or lower concentrations, see methods for exceptions) could inhibit biofilm formation in the presence of subinhibitory concentrations of the commonly-prescribed antifungal agents amphotericin B, caspofungin, and fluconazole (see methods). We found that ten of the 43 compounds inhibited biofilm formation in the sustained inhibition biofilm assay when combined with one or more of the three commonly prescribed antifungal agents (Figure 2c and Figure S3b–d, File S4). Five compounds inhibited biofilm formation in the presence of caspofungin, one inhibited biofilm formation in the presence of amphotericin B, one inhibited biofilm formation in the presence of fluconazole, two inhibited biofilm formation in the presence of either amphotericin B or caspofungin, and one inhibited biofilm formation in the presence of amphotericin B, caspofungin, or fluconazole (Figure 2d, File S4). Three of the ten compounds identified in the combination inhibition biofilm assays did not exhibit effects in the stand-alone secondary screen at 40 µM; these were the thyroid hormone liothyronine sodium, the antibiotic erythromycin, and the heme precursor protoporphyrin ix.

### 3.4. Disruption Assays

**Figure 3:**
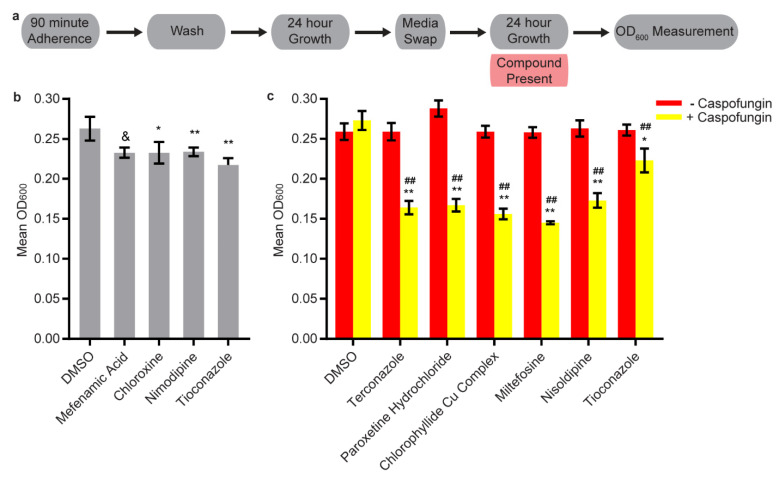
Fourteen candidate compounds disrupted mature biofilms by themselves or in combination with the known antifungal agent caspofungin. (**a**) Overview of the disruption optical density biofilm assay used for these experiments. In brief, the media was removed after the 24-h growth step and fresh media containing the compound was added, after which biofilms were grown in vitro for an additional 24 h. (**b**) Subset of the statistically significant hits from the stand-alone disruption optical density biofilm assay. Mean OD_600_ readings with standard deviations are shown, significant differences from the DMSO solvent control as determined by Welch’s *t*-test (two-tailed, assuming unequal variance) with the Bonferroni correction are indicated for α = 0.05 (*), α = 0.01 (**), or mixed results (&). Although a single repeat is shown, the indicated significance threshold was met by all of the repeats of each compound shown with the exception of mefenamic acid. In that case, one of the four repeats did not pass either significance threshold while the remaining three repeats passed at α = 0.01. (**c**) Subset of the statistically significant hits from the combination disruption optical density biofilm assays with caspofungin. For each compound, the wells with caspofungin (+ caspofungin) are indicated in yellow and the wells without caspofungin (− caspofungin) are indicated in red. Mean OD_600_ readings with standard deviations are shown; significant differences from the compound without caspofungin control (e.g., nisoldipine, − caspofungin), as determined by Welch’s *t*-test (two-tailed, assuming unequal variance) with the Bonferroni correction, are indicated for α = 0.05 (*) and α = 0.01 (**). Significant differences from the caspofungin without compound control (e.g., DMSO, + caspofungin), as determined by Welch’s *t*-test (two-tailed, assuming unequal variance) with the Bonferroni correction are indicated for α = 0.05 (#) and α = 0.01 (##). In both panels b and c, the data within a chart are all taken from the same plate on the same day.

Because the ability to inhibit biofilm formation does not necessarily translate into the ability to disrupt a mature biofilm, we tested the 43 candidate compounds at 40 µM for effects in the disruption optical density biofilm assay (Figure 3a). Four compounds were able to disrupt mature biofilms by themselves (Figure 3b, File S4); two of these also had effects in either the stand-alone or combination sustained inhibition biofilm assays (chloroxine and tioconazole). Three of these four compounds also had effects at 40 µM in the low throughput adherence inhibition assay (chloroxine, nimodipine, tioconazole). Two of the four compounds (chloroxine and tioconazole) had known antimicrobial activities; to our knowledge the other two compounds (the anti-inflammatory agent mefenamic acid and the calcium channel blocker nimodipine) had not been previously recognized as antifungals. Both mefenamic acid and nimodipine appear to work preferentially against biofilms as they did not inhibit planktonic growth at concentrations of at least 200 µM (Table 1).

We next tested the 43 candidate compounds at 12.5 µM or lower concentrations (see methods for exceptions) for their abilities to disrupt mature biofilms in combination with sub-disruptive concentrations of amphotericin B, caspofungin, or fluconazole (see methods). Eleven compounds disrupted biofilms in the presence of caspofungin (Figure 3c and Figure S4, File S4), seven of which also had combination effects in the sustained inhibition biofilm assay. Among the compounds that had synergistic effects in both assays are liothyronine sodium, nisoldipine, and paroxetine hydrochloride (Figure 3c and Figure S4, File S4).

## 4. Discussion

**Figure 4:**
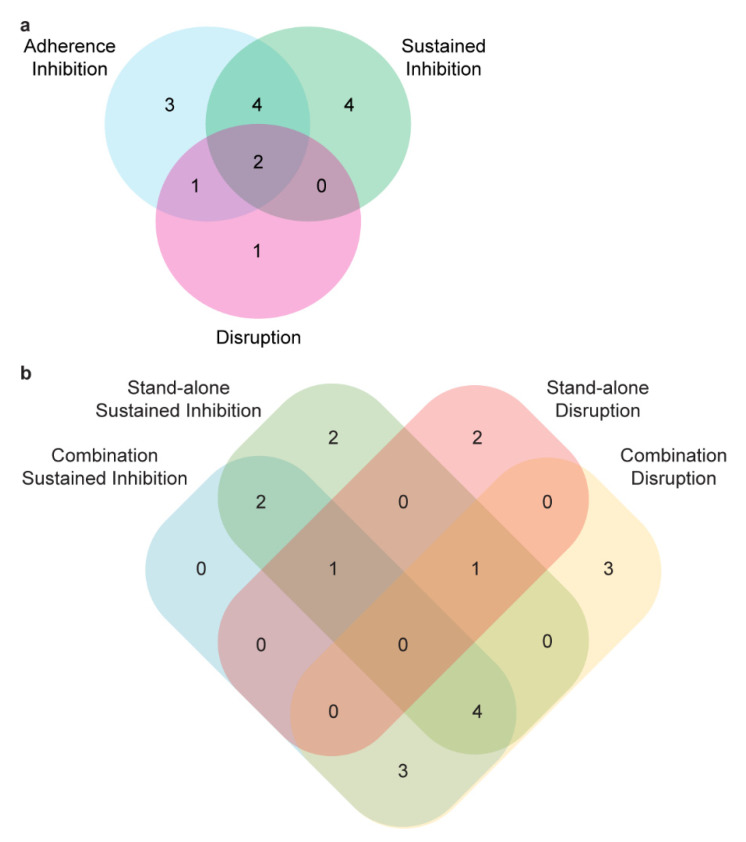
A number of compounds had effects in only a subset of the biofilm assays. (**a**) Compounds with an effect by themselves at 40 µM in the adherence inhibition, sustained inhibition, or disruption optical density biofilm assays are indicated. In total, 14 compounds had an effect in at least one of these three assays. (**b**) Compounds with an effect in either the stand-alone or the combination versions of the sustained inhibition or disruption optical density biofilm assays are indicated. In total, 18 compounds had an effect in at least one of these four assays.

The opportunistic human fungal pathogen *Candida albicans* can form biofilms on tissues and implanted medical devices, posing serious health concerns. Only three classes of drugs are currently used to treat fungal infections in humans, and it remains a challenge to develop new antifungal drugs. The ability to “repurpose” drugs significantly expedites drug discovery because the pharmacology and toxicology of the drug have already been established [59]. In this study, we report several FDA approved compounds effective at inhibiting biofilm formation or disrupting mature biofilms in vitro either by themselves or in combination with one or more commonly used antifungal agents. Multiple calcium channel blockers, a selective serotonin reuptake inhibitor, an inhibitor of viral DNA replication, an anticoagulant that inhibits thrombin, and an azole-based proton pump inhibitor all showed antibiofilm effects in at least one of our assays. Based on the proof of concept results we present here, the next step in exploring the antibiofilm properties of these compound classes is to test additional derivatives from these classes for antifungal and antibiofilm properties. Identified compounds and their derivatives that are able to disrupt mature *C. albicans* biofilms may be most useful for treating recurrent infections, while those that are able to inhibit *C. albicans* biofilms may be most useful as a preventative strategy to be given prophylactically to high risk individuals. As might be expected based on the inherent resilience of mature biofilms, we identified only a few compounds that could disrupt mature biofilms either by themselves or in combination with conventional antifungal agents (Figure 4). We also identified several compounds that did not affect planktonic *C. albicans* growth but were able to inhibit biofilm formation or disrupt mature biofilms. These compounds are potential biofilm-specific drug candidates, which are currently not known to exist for treating *C. albicans* infections. These compounds could, for example, impact the production or the extracellular matrix, cell-cell or cell-surface adherence during biofilm formation, cell-cell communication within a biofilm, or a number of other known and unknown biofilm processes that do not occur in the planktonic cell state.

Although we detected one compound, artemisinin, that was also identified in the two previously published screens of this library [41,42], most of our results differed from previous reports, underscoring the degree to which the experimental setup may affect the outcome. Consistent with this idea, the results of our stand-alone and combination screens as well as our adherence inhibition, sustained inhibition, and disruption assays did not fully overlap; rather there were hits unique to each of these assays (Figure 4). Clearly, conducting multiple screens of a compound library with differences in the experimental approach and the compounds tested for synergy is useful to maximize the chances of finding existing therapeutics that could be repurposed as antifungal agents.

## Figures and Tables

**Table 1:** Planktonic MICs and sustained inhibition optical density biofilm assay MBICs of selected compounds.

Compound	Shorthand Code	MBIC (µM)	MIC (µM)
disulfiram	PH04	50	25
mefenamic acid	PH12	50	>200
artemisinin	PH17	50	>200
chloroxine	PH20	50	25
terconazole	PH25	0.8	≤0.2
dabigatran etexilate mesylate	PH26	200	>200
paroxetine hydrochloride	PH27	50	>200
dexlansoprazole	PH28	3.1	>200
miltefosine	PH33	50	12.5
nisoldipine	PH36	50	>200
flucytosine	PH37	50	>200
tioconazole	PH38	0.8	≤0.2

## Supplementary Materials

The following are available online at https://www.mdpi.com/2076-2607/8/5/756/s1, **Figure S1**. Comparisons of the B-scores for each of the 1600 compounds tested in the two high-throughput adherence inhibition optical density biofilm assays. The 43 candidate hits that were pursued further are indicated in red. The 27 other hits that were not pursued further, which consist of a mixture of well-known antifungal agents, compounds intended for topical use, and compounds with high toxicity values (<100 mg/kg), are indicated in blue. All other compounds are indicated in black. The dotted lines indicate a B-score of zero and the dashed lines indicate a B-score of minus four. **Figure S2.** Additional hits from the adherence inhibition optical density biofilm assay. In panels a, b, d, and e, mean OD_600_ readings with standard deviations are shown, significant differences from the DMSO solvent control as determined by Welch’s *t*-test (two-tailed, assuming unequal variance) with the Bonferroni correction are indicated for α = 0.05 (*) and α = 0.01 (**). (a) Hits at 10 µM in the adherence inhibition optical density biofilm assay. (b) Further hits at 40 µM in the adherence inhibition optical density biofilm assay. (c) Comparison of the mean normalized absorbance (OD_600_) for the 10 µM and 40 µM adherence inhibition optical density biofilm assays. Compounds that were hits at 10 µM are indicated in blue, at 40 µM are indicated in red, and all other compounds are indicated in black. Both hits at 10 µM were also hits at 40 µM. The mean absorbance for each compound was normalized to the mean absorbance for all DMSO loading control wells from this experiment. (d) Adherence inhibition optical density biofilm assay results at 40 µM for the four compounds that were hits in the sustained inhibition optical density biofilm assay at 40 µM but that were not hits in the adherence inhibition optical density biofilm assay. (e) Sustained inhibition optical density biofilm assay results at 40 µM for the four compounds that were hits in the adherence inhibition optical density biofilm assay at 40 µM but were not hits in the sustained inhibition optical density biofilm assay. Mixed statistical results between different replicates of the sustained inhibition optical density biofilm assay are indicated with a %, these compounds each had one statistically significant result out of the two or four times, respectively, they were tested in this assay. **Figure S3**. Additional hits from the stand-alone and combination sustained inhibition optical density biofilm assays. (a) Statistically significant hits from the stand-alone sustained inhibition optical density biofilm assay. Mean OD_600_ readings with standard deviations are shown; significant differences from the DMSO solvent control as determined by Welch’s *t*-test (two-tailed, assuming unequal variance) with the Bonferroni correction are indicated for α = 0.05 (*) and α = 0.01 (**). Although a single repeat is shown, the indicated significance threshold was met by all of the repeats of each compound shown. (b) Statistically significant hits from the combination sustained inhibition optical density biofilm assays with amphotericin B. For each compound, the wells with amphotericin B (+ amphotericin B) are indicated in orange and the wells without amphotericin B (− amphotericin B) are indicated in green. (c) Statistically significant hits from the combination sustained inhibition optical density biofilm assays with fluconazole. For each compound, the wells with fluconazole (+ fluconazole) are indicated in purple and the wells without fluconazole (− fluconazole) are indicated in blue. (d) Statistically significant hits from the combination sustained inhibition optical density biofilm assays with caspofungin. For each compound, the wells with caspofungin (+ caspofungin) are indicated in yellow and the wells without caspofungin (− caspofungin) are indicated in red. In panels b–d, mean OD_600_ readings with standard deviations are shown, significant differences from the compound without antifungal agent controls (e.g., chloroxine, − amphotericin B), as determined by Welch’s *t*-test (two-tailed, assuming unequal variance) with the Bonferroni correction, are indicated for α = 0.05 (*) and α = 0.01 (**). Significant differences from the antifungal agent without compound control (e.g., DMSO, + amphotericin B), as determined by Welch’s *t*-test (two-tailed, assuming unequal variance) with the Bonferroni correction, are indicated for α = 0.05 (#) and α = 0.01 (##). In panels a–c, the data within each panel are all taken from the same plate on the same day. In panel d, data from separate plates are separated by two vertical lines on the x-axis; the DMSO solvent control is shown for each plate. **Figure S4**. Additional hits from the combination disruption optical density biofilm assays with caspofungin. For each compound, the wells with caspofungin (+ caspofungin) are indicated in yellow and the wells without caspofungin (− caspofungin) are indicated in red. Mean OD_600_ readings with standard deviations are shown; significant differences from the compound without caspofungin control (e.g., zolpidem, − caspofungin), as determined by Welch’s *t*-test (two-tailed, assuming unequal variance) with the Bonferroni correction, are indicated for α = 0.05 (*) and α = 0.01 (**). Significant differences from the caspofungin without compound control (e.g., DMSO, + caspofungin), as determined by Welch’s *t*-test (two-tailed, assuming unequal variance) with the Bonferroni correction, are indicated for α = 0.05 (#) and α = 0.01 (##). Data from separate plates are separated by two vertical lines on the x-axis; the DMSO solvent control is shown for each plate. **File S1**. Screen of the Pharmakon 1600 compound library for compounds with the ability to inhibit *C. albicans* biofilm formation in the adherence inhibition optical density biofilm assay. Differences from the mean (in units of standard deviation) and the B-score for all compounds from both screens of the library (at a concentration of 10 µM) are provided. **File S2**. Identities of the 70 candidate compounds identified based on the initial adherence inhibition optical density biofilm assay and the basis for excluding 27 of these compounds from further consideration. Differences from the mean (in units of standard deviation) and the B-score from both screens of the library are indicated for these compounds. **File S3:** Supplementary Materials and Methods. Details on the statistical analyses for the low throughput adherence inhibition screen (2.5), sustained inhibition and disruption testing (2.6), MBIC determination (2.7), and combination screening (2.9). **File S4**. Summary of the hits from the 10 µM and 40 µM low throughput adherence inhibition, the stand-alone and combination sustained inhibition, and stand-alone and combination disruption optical density biofilm assays. **File S5**. Compiled data and statistics from the 10 µM and 40 µM low throughput adherence inhibition, the stand-alone and combination sustained inhibition, and the stand-alone and combination disruption optical density biofilm assays as well as the BIC sustained inhibition optical density biofilm assay. For each compound, the concentration used, average OD_600_, average OD_600_ of relevant control(s), and value(s) for Welch’s *t*-test versus the relevant control(s) are provided. Whether the average OD_600_ was below the average OD_600_ of the relevant control(s) and whether the difference from the relevant control(s) remains significant following the Bonferroni correction (α = 0.05) are also indicated. This file also contains the sensitivity of SNY425 to amphotericin B, caspofungin, and fluconazole in our biofilm assays. **File S6**. Compiled data for the planktonic MIC assays. The average OD_600_ for the two replicates is shown for each compound at each concentration.
