# Supplementary material for: A Selective Serotonin Reuptake Inhibitor, a Proton Pump Inhibitor, and Two Calcium Channel Blockers Inhibit Candida albicans Biofilms
Source: Microorganisms. 2020 May 18;8(5):756. doi: 10.3390/microorganisms8050756 (PMC7285287; doi:10.3390/microorganisms8050756)
Supplement: Supplementary file 1 [file microorganisms-08-00756-s001.zip › Nobile et al File S3.docx]

**A selective serotonin reuptake inhibitor, a proton pump inhibitor, and two calcium channel blockers inhibit *Candida albicans* biofilms**

Clarissa J. Nobile^1^, Craig L. Ennis^1,2^, Nairi Hartooni^3,#^, Alexander D. Johnson^3,4^, Matthew B. Lohse^3,5,*^

**File S3:** Supplementary Materials and Methods. Details on the statistical analyses for the low throughput adherence inhibition screen (2.5), sustained inhibition and disruption testing (2.6), MBIC determination (2.7), and combination screening (2.9).

**S2. Supplementary Materials and Methods**

*S2.5 Low Throughput Adherence Inhibition Screen*

In order to correct for the multiple comparisons performed, the Bonferroni Correction with α = 0.05 was applied. All of the comparisons for a given concentration (e.g. all of the 10 µM or 40 µM tests) were pooled for the multiple comparisons correction step, giving a number of hypotheses, m, of 43 for each concentration (for a final threshold of 1.16 x 10^-3^). We then determined whether each experimental repeat (1) had an average absorbance less than the average of the control wells (both on the plate the repeat was on and for the control wells across all of the plates) and (2) was significant after the multiple comparisons correction.

*S2.6 Sustained Inhibition and Disruption Testing*

In order to correct for the multiple comparisons performed, the Bonferroni Correction with α = 0.05 was applied. All of the comparisons for a given type of assay (e.g. all of the stand-alone Sustained Inhibition Optical Density Biofilm Assays) were pooled for the multiple comparisons correction step, giving a number of hypotheses, m, of 116 for the Sustained Inhibition Optical Density Biofilm Assay and of 118 for the Disruption Optical Density Biofilm Assay (for final thresholds of 4.31 x 10^-4^ and 4.24 x 10^-4^ respectively). We then determined whether each experimental repeat (1) had an average absorbance less than the average of the control wells and (2) was significant after the multiple comparisons correction. To be considered a validated “hit”, a compound had to satisfy both of these criteria in each repeat if it was tested twice, for at least two repeats if it was tested three times, and for at least three repeats if it was tested four times.

*S2.7 MBIC Determination*

Statistical testing was performed as described for the Sustained Inhibition and Disruption Testing with the following exceptions. The significance was evaluated for a given concentration of compound (e.g. 50 µM nisoldipine) versus the equivalent DMSO loading control (e.g. the mock 50 µM DMSO loading control wells). All of the comparisons, regardless of compound concentration, were then pooled for the multiple comparisons correction step, giving a number of hypotheses, m, of 80 (for a final threshold of 6.25 x 10^-4^). We then determined whether each concentration of a drug (1) had an average absorbance less than the average of the relevant control wells and (2) was significant after the multiple comparisons correction. The MBIC of a compound was defined as the lowest concentration that met these requirements (there were no instances where a higher concentration of a compound failed to also meet these requirements). If no concentration met these requirements, the MBIC is indicated as greater than 200 µM (the highest concentration tested). If all concentrations for a compound met these requirements, the MBIC is indicated as less than or equal to 0.78 µM (the lowest concentration tested).

*S2.9 Combination Screening*

Statistical analysis was performed using Welch’s t-test and the Bonferroni Correction as described for the Sustained Inhibition and Disruption Testing with the following modifications. Each experimental condition was compared to both the relevant antifungal agent control and the relevant candidate control (e.g. a nisoldipine plus caspofungin experiment was compared to the nisoldipine-only control and the caspofungin-only control from the same plate). All of the same comparisons for a given assay (e.g. all of the antifungal agent comparisons for the combination Sustained Optical Density Biofilm Inhibition Assay) were pooled for the multiple comparisons correction step, giving a number of hypotheses, m, of 126 for both the antifungal agent and candidate comparisons in both the Sustained Inhibition and Disruption Optical Density Biofilm Assays (final threshold, 3.97 x 10^-4^). To be considered a validated combination hit, a given experimental condition had to (1) have an average absorbance less than the averages of both sets of relevant control wells and (2) remain significant after the multiple comparisons correction for both sets of comparisons.
