# Supplementary figures and images for: A Selective Serotonin Reuptake Inhibitor, a Proton Pump Inhibitor, and Two Calcium Channel Blockers Inhibit Candida albicans Biofilms
Source: Microorganisms. 2020 May 18;8(5):756. doi: 10.3390/microorganisms8050756 (PMC7285287; doi:10.3390/microorganisms8050756)

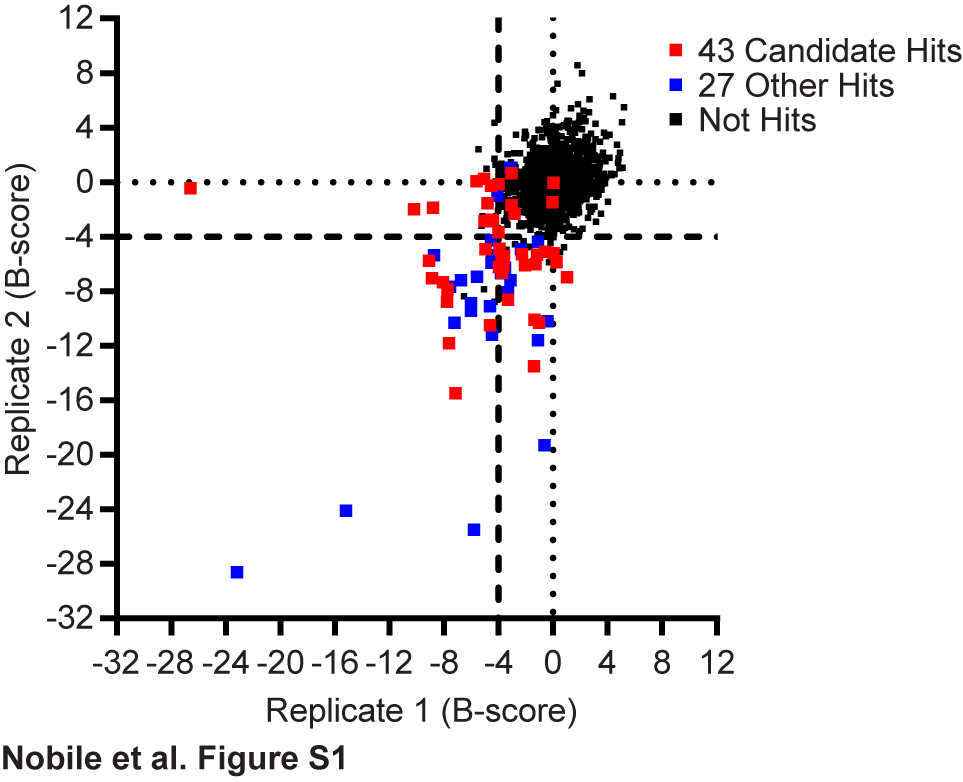

Supplement: Supplementary file 1 [file microorganisms-08-00756-s001.zip › Nobile et al Figure S1.tif]

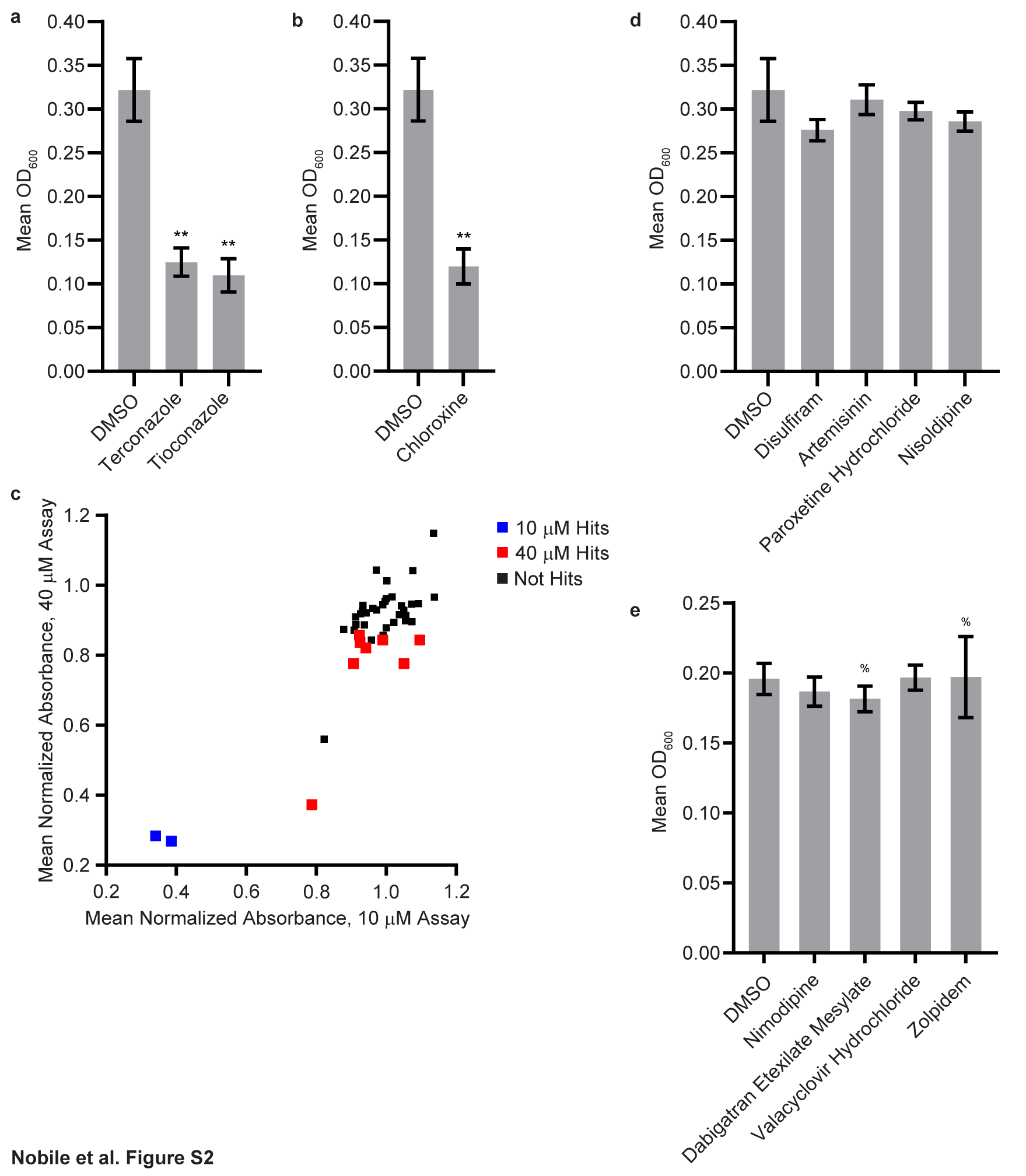

Supplement: Supplementary file 1 [file microorganisms-08-00756-s001.zip › Nobile et al Figure S2.tif]

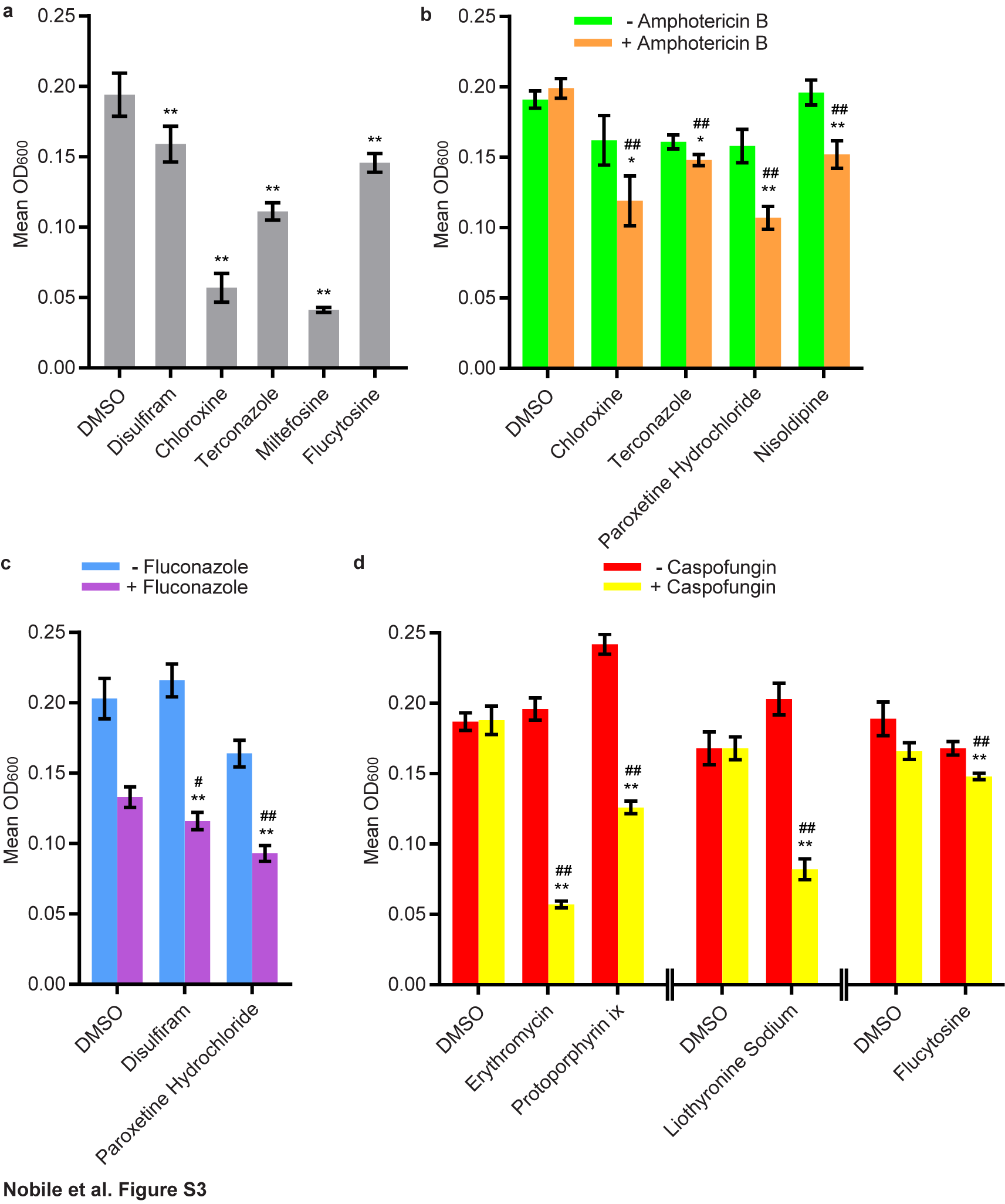

Supplement: Supplementary file 1 [file microorganisms-08-00756-s001.zip › Nobile et al Figure S3.tif]

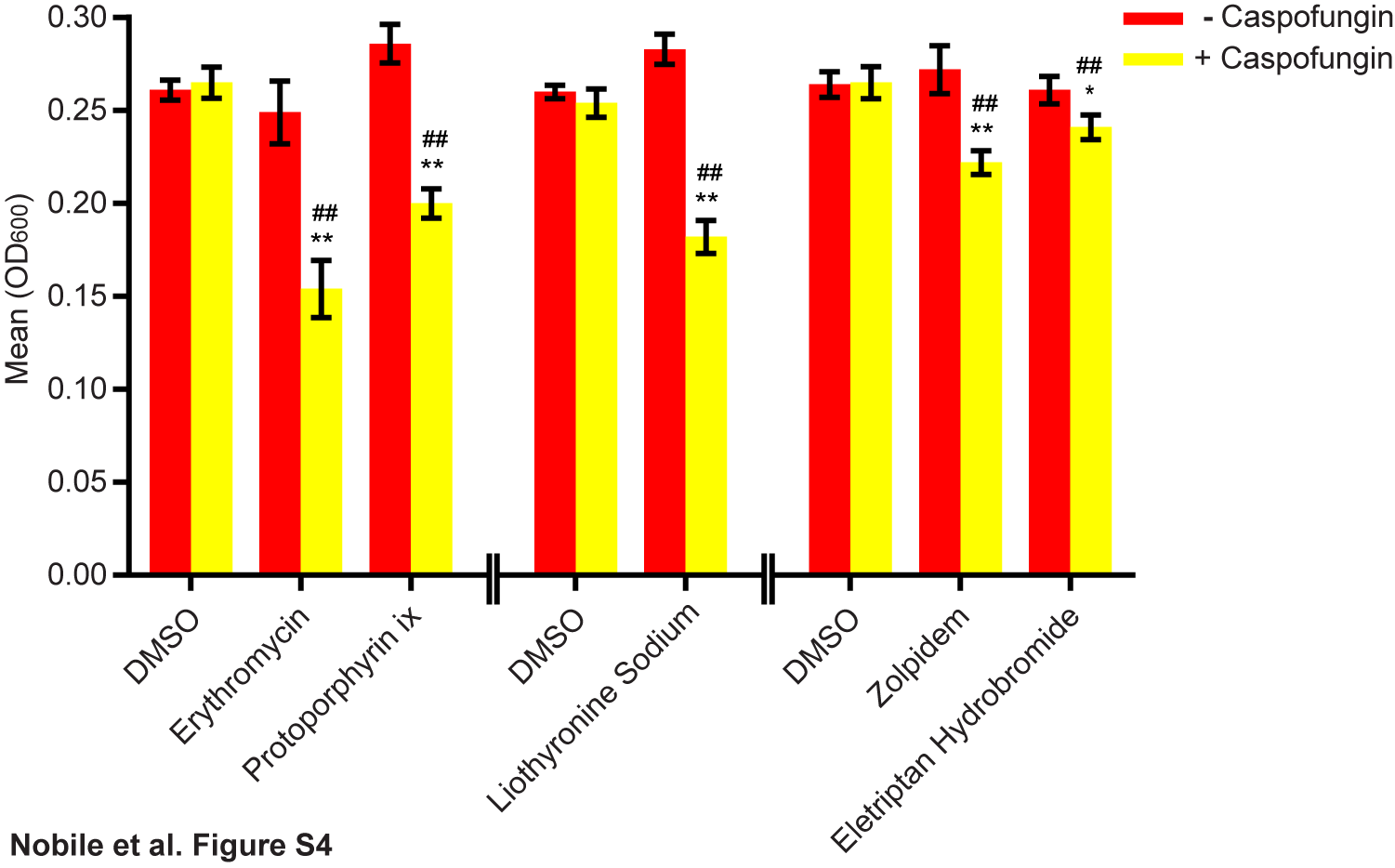

Supplement: Supplementary file 1 [file microorganisms-08-00756-s001.zip › Nobile et al Figure S4.tif]
